# Our Title Page

**Published:** 1884-03

**Authors:** 


### OUR TITLE PAGE.

The title page of THE JOURNAL is fitly ornamented with a likeness of Dr. Crawford W. Long, the discoverer of anæsthesia, the greatest benefactor to the human race which this country or this age has produced.

The history of the picture from which this engraving is taken is interesting. It will be remembered that priority of the use of sulphuric ether as an anæsthetic agent was claimed by a firm of dentists in Boston, who attempted to use it, under the name of lethion, as a secret remedy in their business. Subsequently an application was made to Congress for a reward of one hundred thousand dollars to them as the original discoverers of its value. This application was referred to a committee, before whom Dr. Long appeared and submitted the history of his discovery, and indisputable proofs of his producing anæsthesia with it and his performance of surgical operations without pain while the subjects were under its influence, several years prior to its employment by the Boston claimants.

Their application for reward as the original discoverers, of course, failed.

Dr. Long's account of the first suggestion of it to his own mind, of his cautious and painstaking experiments, and the final operation in 1842, by which he demonstrated its beneficent power, was published to the profession with characteristic modesty.

The use and reputation of ether began to spread throughout the world when the discovery of the value of chloroform by Dr. Simpson, of Edinborough, for like purposes was announced, and for a time supplanted the ether in practice. The question of the original discovery of the anæsthetic properties of the latter temporarily lost much of its interest.

Frequent fatal results happening after the use of chloroform again turned professional attention to the older and safer anæsthetic.

At an opportune moment, Dr. Marion Sims published anew the history of Dr. Long's experiments and a synopsis of his conclusive evidence of priority in discovery and use.

This publication attracted the general attention of scientific men everywhere. Among others Mr. Stewart, of New York, deeply impressed with the priceless value of the discovery to humanity, and the modesty and genius of the discoverer, sought to tender his individual homage to both by procuring at his own expense a magnificent picture of Dr. Long, and presenting it to the State of his nativity.

The General Assembly, being in session, gratefully received the gift, and ordered it hung in the Representative Hall among the portraits of the distinguished dead who have illustrated the history of Georgia.

The inauguration ceremonies on the occasion of the unveiling of the portrait in the Capitol were grand and imposing. Both branches of the General Assembly, his Excellency the Governor, and the State officials, were in attendance, together with a vast throng of the brilliant and cultivated of the community. Choice gems of eloquence celebrated the fame and labors of the great benefactor, touching words of eulogy, expressing the deepest love and regard for the memory of the good man, "the beloved physician."

To make the tribute to his memory more permanent and more valuable, the General Assembly unanimously passed the subjoined resolutions, indissolubly connecting the names of Crawford W. Long and General James Oglethorpe, the two greatest heroes of benevolence which Georgia's annals furnish:

WHEREAS, It has been proposed that each State of the United States of America should designate the names of two persons whose memories are to be perpetuated by likenesses in statuary, in the Art Gallery, established, or to be established in Washington City, the Federal Capital; *and whereas*, anæsthesia is the greatest boon ever conferred upon humanity, unless vaccination claims equal title to be so considered; *and whereas*, Crawford W. Long, M.D., a native of Georgia, and graduate of the University of the State, lately deceased, is the historic discoverer of anæsthesia, and the first man to employ sulphuric ether as an anæsthetic agent in a surgical operation on the 30th of March, 1842; *and whereas*, England recognized the labors of Jenner, and also bestowed a high honor of government upon Sir James Y. Simpson, in recognition of the great service he had rendered humanity by the introduction of chloroform, which enlarged the domain of anæsthesia; *and whereas*, our Federal Republic should not allow the names of our discoverers and scientists to rest in obscurity, and the State of Georgia should especially cherish with pride the name and memory of her great discoverer:

*Resolved by the General Assembly*, That the name of Crawford W. Long, M.D., the historic discoverer of anæsthesia, be presented to the Art Gallery at Washington City, established, or to be established, to represent the State of Georgia.

*Resolved*, That the name of James Oglethorpe, the historic founder of the Commonwealth of Georgia, be also presented to said gallery to represent the State.

*Resolved*, That a copy of this preamble and resolutions be transmitted by the Governor to his Excellency, the President of the United States of America, with a request that he submit them to the Senate and House of Representatives of Congress on its next assemblage, and that another copy thereof be sent by the Governor to the proper officer in charge of said Art Gallery.

Approved August 23, 1879.

